# Fulminant hepatic failure in a patient testing re-positive for SARS-CoV-2: a case report

**DOI:** 10.1186/s12245-021-00349-6

**Published:** 2021-04-21

**Authors:** Bader Aldossary, Ali Hassan, Mohamed Moussa, Hind S. Alsaif, Dunya Alfaraj

**Affiliations:** 1 Department of Emergency Medicine, King Fahd Hospital of the University, Imam Abdulrahman Bin Faisal University, Al-Khobar, Saudi Arabia; 2 Department of Radiology, Salmaniya Medical Complex, Manama, Bahrain; 3 Department of Radiology, King Fahd Hospital of the University, Imam Abdulrahman Bin Faisal University, Al-Khobar, Saudi Arabia

**Keywords:** Case reports, COVID-19, Liver failure, Re-positive test, Re-infection

## Abstract

**Background:**

Infection by severe acute respiratory syndrome coronavirus 2 (SARS-CoV-2) may not elicit lifelong protective immunity and reinfection could occur. Liver function impairment is a common manifestation of coronavirus disease 2019 (COVID-19). However, acute hepatic failure in the setting of COVID-19 is very rare.

**Case presentation:**

We report the case of a 47-year-old woman who presented with acute abdominal pain and vomiting. Abdominal examination revealed a soft and lax abdomen with mild tenderness in the right upper quadrant. The patient recovered from COVID-19 2 months previously with negative results on reverse transcription-polymerase chain reaction (RT-PCR). Laboratory investigations revealed markedly elevated transaminases with normal results on viral hepatitis serology panel and undetectable blood paracetamol level. Prior to admission, the patient underwent RT-PCR for SARS-CoV-2, which revealed a positive result. The patient experienced rapid deterioration in the neurological status with a remarkable increase in the liver enzyme levels. Despite aggressive resuscitation, the patient suffered irreversible cardiac arrest and died.

**Conclusion:**

Fulminant hepatic failure is a rare manifestation in patients with re-positive RT-PCR tests for SARS-CoV-2. Clinicians should maintain a high index of suspicion for hepatic injury with active monitoring of liver enzymes.

## Background

Coronavirus disease 2019 (COVID-19) is a highly transmissible infection caused by severe acute respiratory syndrome coronavirus 2 (SARS-CoV-2). It became evident that the infection by SARS-CoV-2 might not elicit lifelong protective immunity and, therefore, reinfection could occur [1]. While it is suggested that the initial infection could attenuate the severity of reinfection [2, 3], some cases demonstrated that reinfection may result in a more severe clinical event [1, 4].

The spectrum of clinical severity of COVID-19 is very broad, with most symptomatic cases having a mild course [5]. The clinical manifestations of COVID-19 have been characterized by cough, fever, myalgia, and headache [5]. Moreover, extrapulmonary involvement has been frequently observed. It is estimated that up to 60% of cases have liver function impairment [6], which mainly manifests as elevated levels of aminotransferases [7]. Acute hepatic failure, however, is an extremely rare condition in patients with COVID-19 [8]. Here, we report a middle-aged woman who developed a fulminant hepatic failure in the setting of a probable COVID-19 reinfection who presented with acute abdominal pain without pulmonary symptoms.

## Case presentation

We present the case of a 47-year-old woman, with no underlying clinical conditions, who presented to the emergency department with a 2-day history of severe right upper quadrant abdominal pain radiating to the right shoulder. She described the pain as sharp and constant. Her pain was associated with recurrent episodes of vomiting. The patient reported taking paracetamol and an herbal product, which did not result in any improvement. She could not identify the ingredients of the herbal preparation, but she reported using it regularly for its analgesic properties. She does not drink alcohol and has never smoked. There is no history of liver diseases in the family.

On examination, her vital signs were within normal limits and the abdominal examination revealed a soft and lax abdomen with mild tenderness in the right upper quadrant. Two months prior to presentation, the patient experienced mild respiratory tract symptoms and she tested positive for severe acute respiratory syndrome coronavirus 2 (SARS-CoV-2). She had negative results on two consecutive reverse transcription-polymerase chain reaction (RT-PCR) tests after 2 weeks of home isolation considering her mild symptoms with no derangements in laboratory parameters.

**Fig. 1:**
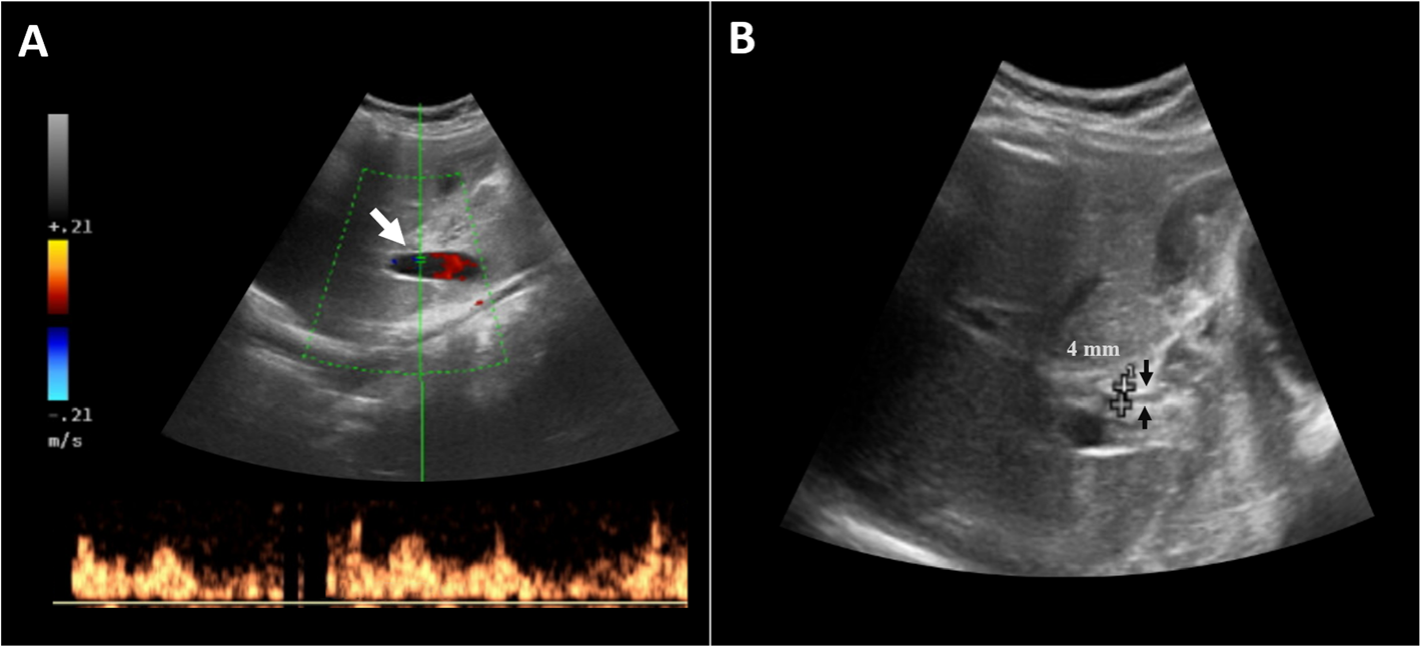
Abdominal ultrasound image showing normal hepatic echogenicity with hepatopetal flow in portal vein (arrow in **a**) and non-dilated common bile duct (arrows in **b**)

Abdominal ultrasound demonstrated the liver with smooth capsular contour and normal parenchymal echogenicity. It had a normal hepatopetal blood flow in the portal vein with no evidence of biliary duct obstruction (Fig. 1). Laboratory findings revealed markedly elevated hepatic enzymes and deranged coagulopathy profile. However, the viral serology, autoimmune, toxicology profiles were negative (Table 1).

**Table 1:** Summary of the results of laboratory findings on presentation

Laboratory investigation		Result	Reference range
**Hematological profile**	Hemoglobin (g/dL)	11.4	13.0–18.0
	White blood cell (1000/μL)	9.8	4.0–11.0
	Platelet (1000/μL)	448	140–450
	Neutrophils (%)	75.4	40–60
	Lymphocytes (%)	11.0	20–40
**Hepatic profile**	Total bilirubin (mg/dL)	1.3	0.2–1.2
	Albumin (g/dL)	4.0	3.4–5.0
	Alkaline phosphatase (U/L)	150	46–116
	γ-Glutamyltransferase (U/L)	49	15–85
	Alanine transferase (U/L)	2,411	14–63
	Aspartate transferase (U/L)	2,466	15–37
**Coagulation profile**	Prothrombin time (s)	>120	12.9–15.9
	Partial thromboplastin time (s)	120	25.6–42.3
	International normalized ratio	>15.8	0.8–1.1
**Inflammatory profile**	C-Reactive protein (mg/dL)	<0.10	0.10–0.50
	Lactic acid (mmol/L)	19.7	0.5–2.2
	Ferritin (ng/ml)	165.2	4.6–204.0
**Serology profile**	Hepatitis A IgM antibody	Negative	Negative
	Hepatitis B core antibody	Negative	Negative
	Hepatitis B surface antibody	Negative	Negative
	Hepatitis C virus antibody	Negative	Negative
	Ebstein-Barr virus IgM antibody	Negative	Negative
	Cytomegalovirus IgM antibody	Negative	Negative
	Human immunodeficiency virus antibody	Negative	Negative
**Autoimmune profile**	Antimitochondrial antibody	Negative	Negative
	Antinuclear antibody	Negative	Negative
	Anti-liver/kidney microsomal antibody	Negative	Negative
	Anti-smooth-muscle antibody	Negative	Negative
**Toxicology profile**	Acetaminophen	Negative	Negative
	Tetrahydrocannabinol	Negative	Negative
	Benzodiazepines	Negative	Negative

**Fig. 2:**
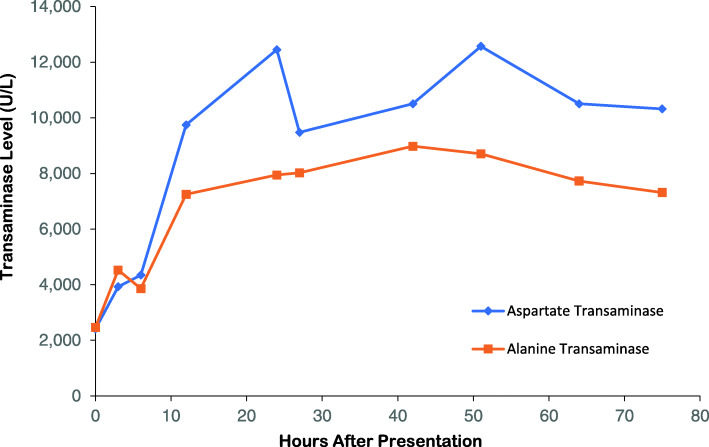
Levels of liver enzymes over time

In light of the aforementioned clinical and laboratory findings, the patient was admitted to the intensive care unit for monitoring and continued supportive care. As per local institutional policy, the patient underwent RT-PCR for SARS-CoV-2, which revealed a positive result. However, the chest radiograph findings were normal. The patient developed rapid deterioration in the neurological status with a remarkable increase in the liver enzyme levels (Fig. 2). The need for emergency liver transplantation was discussed. However, despite aggressive resuscitation, the patient suffered irreversible cardiac arrest and died. An autopsy examination was not performed.

## Discussion

To the best of our knowledge, we described the first case report of a fatal hepatic failure in the setting of COVID-19 that occurred around 2 months after the recovery from the initial infection with negative results on RT-PCR. It should be remembered that false-negative laboratory results and prolonged viral shedding are alternative explanations for a re-positive RT-PCR test [9]. Thus, genomic sequence analysis is essential to confirm the re-infection [10], which was not performed in the present case.

Liver function impairment has been shown to be a predictor of clinical deterioration in patients with COVID-19 [11, 12]. Several mechanisms have been proposed to explain the pathogenesis of liver injury in patients with COVID-19. The hepatic injury in COVID-19 could be related to a direct viral attack. Liver histology in patients with COVID-19 reveals microvascular steatosis with focal necrosis and mild portal and lobular activity [13]. It is postulated that the cellular entry of SARS-CoV-2 is mediated by the angiotensin-converting enzyme 2 (ACE2) [14]. However, transaminases are more commonly elevated than alkaline phosphatase despite that ACE2 is predominantly expressed on cholangiocytes than hepatocytes [15, 16]. There has been some concern that about the role of ACE inhibitors in the virulence of SARS-CoV-2 by upregulating the ACE2 receptors [17]. In the present case, however, the patient was not on any ACE inhibitors.

Patients with COVID-19 may experience cytokine storm syndrome, with the excessive release of inflammatory mediators due to overactivation of the immune system [18]. Li et al. [19] showed that elevated levels of C-reactive protein serve as a predictor of liver injury. Alternatively, hypoxemia from COVID-19 can result in metabolic dysregulation in multiple organs, including the liver, leading to hepatocyte death [20]. However, in the present case, the patient did not show any pulmonary involvement in the reinfection event.

Considering that fever is a common presentation of COVID-19, patients may use antipyretic medications, including paracetamol, which is a known cause of hepatic injury. In the present case, the patient did not report using a significantly large amount of paracetamol and blood paracetamol level was undetectable. The patient reported using some traditional herbal preparation of unknown ingredients, which could play a role in the rapid progression of the disease [21]. However, it seems less likely that the hepatic disease was directly related to the herbal preparation, as the patient reported regular use of the same dose of this preparation for pain relief since a long time.

## Conclusion

Fulminant hepatic failure is a rare manifestation in patients with a re-positive RT-PCR test for SARS-CoV-2. Clinicians should maintain a high index of suspicion for hepatic injury with active monitoring of liver enzymes. It is essential to remember that patients who recovered from COVID-19 may not be completely protected, as recurrence may occur with a more severe course.

## Data Availability

Not applicable.

## Abbreviations

ACE: Angiotensin-converting enzyme
COVID-19: Coronavirus disease 2019
SARS-CoV-2: Severe acute respiratory syndrome coronavirus 2
RT-PCR: Reverse transcription-polymerase chain reaction
